# Cinnamaldehyde Inhibits *Staphylococcus aureus* Virulence Factors and Protects against Infection in a *Galleria mellonella* Model

**DOI:** 10.3389/fmicb.2016.02052

**Published:** 2016-12-21

**Authors:** Thiago A. F. Ferro, Jéssica M. M. Araújo, Bruna L. dos Santos Pinto, Jéssica S. dos Santos, Eliene B. Souza, Bruna L. R. da Silva, Valderlane L. P. Colares, Tânia M. G. Novais, Clovis M. B. Filho, Carsten Struve, João B. Calixto, Valério Monteiro-Neto, Luís C. N. da Silva, Elizabeth S. Fernandes

**Affiliations:** ^1^Programa de Pós-graduação, Universidade CEUMA São Luís, Brazil; ^2^Universidade Federal de Pernambuco Pernambuco, Brazil; ^3^Statens Serum Institut Copenhagen, Denmark; ^4^Centro de Inovação e Estudos Pré-clínicos Florianópolis, Brazil; ^5^Universidade Federal do Maranhão São Luís, Brazil

**Keywords:** essential oil, cinnamaldehyde, infection, bacterial virulence, *S. aureus*

## Abstract

Bacterial resistance to the available marketed drugs has prompted the search of novel therapies; especially in regards of anti-virulence strategies that aim to make bacteria less pathogenic and/or decrease their probability to become resistant to therapy. Cinnamaldehyde is widely known for its antibacterial properties through mechanisms that include the interaction of this compound with bacterial cell walls. However, only a handful of studies have addressed its effects on bacterial virulence, especially when tested at sub-inhibitory concentrations. Herein, we show for the first time that cinnamaldehyde is bactericidal against *Staphylococcus aureus* and *Enterococcus faecalis* multidrug resistant strains and does not promote bacterial tolerance. Cinnamaldehyde actions were stronger on *S. aureus* as it was able to inhibit its hemolytic activity on human erythrocytes and reduce its adherence to latex. Furthermore, cinnamaldehyde enhanced the serum-dependent lysis of *S. aureus*. *In vivo* testing of cinnamaldehyde in *Galleria mellonella* larvae infected with *S. aureus*, showed this compound improves larvae survival whilst diminishing bacterial load in their hemolymph. We suggest that cinnamaldehyde may represent an alternative therapy to control *S. aureus*-induced bacterial infections as it presents the ability to reduce bacterial virulence/survival without promoting an adaptive phenotype.

## Introduction

Bacterial pathogens have evolved several mechanisms to acquire resistance to drug and hereby survive antibiotic treatment in eukaryotic hosts, including mutations, plasmid acquisition, amongst others (Blair et al., 2015; Lin et al., 2015). In fact, multidrug resistant strains have been observed with increasing frequency and their spreading has been recognized as one of the most alarming issues for the global health system, resulting in high levels of morbidity and mortality (Wilson et al., 2016). Infections caused by staphylococcal and enterococcal are reported as a major problem in hospitalized patients especially those using indwelling medical devices such as urinary catheters, feeding tubes, and peripherally inserted central catheters (Padmavathy et al., 2015; Tong et al., 2015). In order to cause infection, these pathogens produce a range of virulence factors which in turn, promote host tissue damage and contribute to bacterial evasion from the host’s immune response and their subsequent survival in the bloodstream (Bhatty et al., 2015; Thammavongsa et al., 2015; Theilacker et al., 2015). This scenario coupled with a diminished antibiotic pipeline has lead to serious social and economic complications and it has prompted the search of novel compounds and therapies to combat bacterial infections (Barriere, 2015).

The antimicrobial properties of plant-derived products have been tested against several pathogens. Cinnamaldehyde is the predominant active compound found in the cinnamon oil from the stem bark of *Cinnamomum cassia*. It is well-known for its wide spectrum antimicrobial activity at concentrations higher than 500 mg/ml (Chen et al., 2015; Shen et al., 2015; Utchariyakiat et al., 2016). The antimicrobial actions of cinnamaldehyde are related to inhibition of cell division through FtsZ (filamentation temperature sensitive protein Z; Domadia et al., 2007), reduction of energy generation and glucose uptake or expenditure (Gill and Holley, 2004) and effects on bacterial cell membrane permeability and integrity (Gill and Holley, 2004; Shen et al., 2015). Recently, cinnamaldehyde was shown to protect against the systemic inflammatory response syndrome (SIRS) induced by the Gram-negative bacteria cell wall component lipopolysaccharide (LPS) in mice (Mendes et al., 2016). A similar effect was observed when cinnamaldehyde was administered to *Galleria mellonella* infected with *Listeria monocytogenes* (Upadhyay and Venkitanarayanan, 2016).

Previous reports described the antibacterial effects of cinnamaldehyde against *Staphylococcus aureus* (Shen et al., 2015) and *Enterococcus faecalis* (Chang et al., 2001). Although, the ability of this compound to interact with the cell walls of these bacteria is well-studied, little is known of its effects on bacterial virulence, especially when tested at sub-inhibitory concentrations. Anti-virulence strategies have gained attention in the recent years as a novel therapeutic paradigm (Rasko and Sperandio, 2010; Kong et al., 2016). These approaches aim to inhibit the synthesis of bacterial virulence factors that are essential for bacterial survival within the host; thus, making the bacteria less pathogenic and/or decreasing the probability of resistance development rather than targeting bacterial viability (Heras et al., 2015).

Here, we investigated the antimicrobial and anti-virulence properties of cinnamaldehyde against *S. aureus* and *E. faecalis*, including multidrug resistant strains. Additionally, we evaluated the ability of cinnamaldehyde to protect against *S. aureus-*induced infection in *G. mellonella* larvae, an alternative model of bacterial infection.

## Materials and Methods

### Bacterial Strains

All tested bacteria were kindly provided by the bacterial collection sector of the Universidade CEUMA and included: six strains of *S. aureus* (standard strains ATCC 25923 and ATCC 6538; clinical isolates SA01, SA02, SA03, SA04); four strains of *E. faecalis* (standard strain ATCC 19433; clinical isolates EF01, EF02, EF03). Susceptibility to antimicrobials was determined in an automated VITEK^®^ 2 system (BioMérieux Clinical Diagnostics, USA) and data interpretation was performed as recommended by the Clinical Laboratory Standards Institute [CLSI] (2015). The multiple antibiotic resistance (MAR) index was calculated using the formula MAR = x/y, where “x” was the number of antibiotics to which the isolate demonstrated resistance; and “y” was the total number of antibiotics tested. The antibiotic susceptibility profile of each strain is shown at **Table 1**.

**Table 1:** Antibiotic susceptibility profiles of *Enterococcus faecalis* and *Staphylococcus aureus* strains.

Strain	Antibiotic							MAR
	PEN	VAN	OXA	GEN	CLI	CIP	SUT	
*E. faecalis* ATCC 19433	S	S	–	S	–	S	–	0
*E. faecalis* 1	S	S	–	S	–	S	–	0
*E. faecalis* 2	R	S	–	S	–	S	–	0.25
*E. faecalis* 3	R	S	–	R	–	R	–	0.75
*S. aureus* ATCC 25923	S	S	S	S	S	S	S	0
*S. aureus* ATCC 6538	S	S	S	S	S	S	S	0
*S. aureus* 1	S	S	S	S	S	S	S	0
*S. aureus* 2	R	S	R	S	R	S	R	0.50
*S. aureus* 3	R	S	R	S	R	S	R	0.50
*S. aureus* 4	R	S	R	S	R	S	S	0.43

*Experiments were performed three times in duplicate.*

*PEN, penicillin; VAN, vancomycin; OXA, oxacillin; GEN, gentamicin; CLI, clindamycin, CIP, ciprofloxacin; SUT, sulfamethoxazole/trimethoprim. Multiple antibiotic resistance (MAR) index.*

### Antimicrobial Assays

The antimicrobial activity of *trans*-cinnamaldehyde (Sigma-Aldrich^®^; 99% purity) was determined by the microdilution method (Clinical Laboratory Standards Institute [CLSI], 2015). Briefly, each strain was grown on Müeller-Hinton Agar (MHA) plates at 37°C for 24 h, and suspended in saline solution (∼1.5 × 10^8^ CFU/ml). For the determination of minimum inhibitory concentrations (MICs), 10 μl of bacterial suspension (approximately 1.5 × 10^8^ CFU/ml) were incubated in Müeller-Hinton (MH) broth containing cinnamaldehyde at different concentrations (62.5–2,000 μg/ml). Serial dilutions of ciprofloxacin (0.06–256 μg/ml) were used as positive controls, while sterile dimethyl sulfoxide (DMSO; 2% in phosphate-buffered saline; PBS) was used as negative control. Samples were then, incubated for 24 h at 37°C. The MIC was defined as the lowest concentration at which no bacterial growth was observed. For determining the minimum bactericidal concentrations (MBCs), just after the MIC experiments, the cultures were seeded on MHA and incubated for 24 h at 37°C. The MBC corresponded to the lowest concentration of the compound to which no viable bacteria was observed.

### Analysis of Bacterial Tolerance to Drug

In order to investigate whether cinnamaldehyde is able to induce bacterial tolerance to drug, we performed serial passage experiments, using the standard strains of *S. aureus* (ATCC 25923) and *E. faecalis* (ATCC 19433). For this, bacterial suspensions (1 ml, ∼1.5 × 10^8^ CFU/ml) were added to six-well tissue culture plates containing MH broth and sub-inhibitory concentrations (MIC/2) of cinnamaldehyde or ciprofloxacin (positive control). After 24 h at 37°C, the culture growing at one dilution below the MIC was used to inoculate the subsequent passage, and this process was repeated for a total of 10 passages. The compound concentration range of each new passage was based on the MIC calculated for the previous passage. Vehicle-treated bacteria (2% DMSO in PBS) were used as negative controls.

### Anti-biofilm Activity

Biofilm formation was quantified according to the method previously described by Stepanović et al. (2004). For this, 10 μl of bacterial suspension (prepared as described above) were added per well in to a 96-well cell culture plate containing sub-inhibitory concentrations of cinnamaldehyde (MIC/2 and MIC/4) and 200 μl of Luria-Bertani (LB) broth. Vehicle (2% DMSO in PBS)-treated bacteria and broth without bacteria were used as positive and negative controls, respectively. Samples were incubated at 37°C and after 24 h, and then, the wells were washed three times with PBS. Biofilm was stained with 5% crystal violet for 10 min at room temperature, and immediately solubilised with methanol (200 μl, 100%). The absorbance was read at 570 nm. Relative biofilm mass results are expressed as percentage (%) in relation to control (vehicle-treated wells). In a different set of experiments, the effects of cinnamaldehyde on bacterial viability were assessed and calculated by addition of PrestoBlue^®^ reagent (1:10; Life Technologies), according to the manufacturer’s instructions. Cell viability is expressed as absorbance in nm.

### Studies with Human Samples

Blood samples were collected from three healthy volunteers with no recent history of taking either antibiotic or anti-inflammatory drugs, and/or infectious or inflammatory diseases in the last 3 weeks prior to sample collection; after a written informed consent was obtained. The study was reviewed and approved by the Human Research Ethics Committee of the Universidade CEUMA (CEP-UNICEUMA) and was performed in accordance with the Declaration of Helsinki 1975, as revised in 2008.

#### Hemolysis Assay

Samples (2.5 ml of blood) were collected in heparinised tubes and the erythrocytes were immediately isolated by centrifugation at 1,500 rpm for 10 min. After removal of plasma, the erythrocytes were washed three times with PBS (pH 7.4) and then suspended in BHI broth. In parallel, bacterial suspensions were obtained as described for MIC determination (∼1.5 × 10^8^ CFU/ml). Aliquots of 10 μl of each bacterial suspension were added into 200 μl of BHI broth supplemented with human erythrocytes (2%) and incubated with sub-inhibitory concentrations of cinnamaldehyde (MIC/2 and MIC/4) or vehicle (2% DMSO in PBS). After 24 h of incubation at 37°C, the tubes were centrifuged and the supernatant (100 μl/per sample/well) was transferred to a 96-well plate. Absorbance was read at 550 nm and taken as an indicative of hemolytic activity. Results are expressed as percentage (%) in relation to the hemolytic activity of each bacterial strain incubated with vehicle (2% DMSO in PBS; vehicle-controls).

#### Analysis of Bacterial Survival following Incubation with Human Serum

This assay was performed according to the method previously described by Ismail et al. (1988), modified. Blood samples (2.5 ml) were collected in tubes containing no anticoagulant. Serum was separated by centrifugation at 1,500 rpm for 10 min. Aliquots (10 μl/well) of the bacterial suspensions (∼1.5 × 10^8^ CFU/ml) were mixed with 140 μl/well of BHI broth containing sub-inhibitory concentrations of cinnamaldehyde (MIC/2 and MIC/4) or vehicle (2% DMSO in PBS) and 60 μl/well of serum. After incubation for 24 h at 37°C, the absorbance was read at 600 nm and taken as bacterial growth index. Results are expressed as percentage (%) in relation to the bacterial growth registered for each bacterial strain incubated with vehicle (2% DMSO in PBS; vehicle-controls).

### Bacterial Adherence to Latex

As previously described (Chandra et al., 2008), siliconized latex catheter segments (4 mm) were placed into tubes containing 4.5 ml of LB medium with and without sub-inhibitory concentrations of cinnamaldehyde or vehicle (2% DMSO in PBS). Then, 225 μl of bacterial suspension (∼1.5 × 10^8^ CFU/ml) were added to each tube. Tubes were incubated at 37°C for 3 h and then, the latex segments were washed three times with PBS and plated on LB Agar. Following incubation for 24 h, at 37°C, plates were analyzed for CFU counting. The results are expressed as CFU/ml.

### *S. aureus*-Induced Infection in *G. mellonella*

The *in vivo* antimicrobial actions of cinnamaldehyde were evaluated in an *in vivo* model of infection induced by *S. aureus* in *G. mellonella* larvae. Briefly, *G. mellonella* larvae (∼200 mg) were randomly distributed in two experimental groups (*n =* 10/group), and were then infected by injection of 10 μl of bacterial suspension (*S. aureus* ATCC 25923; 1.0 × 10^5^ CFU/ml in PBS) in to the last left proleg. The larvae were incubated at 37°C. After 2 h, the larvae received either cinnamaldehyde at different doses (2.5–5.0 μg /100 mg of larvae) or vehicle (PBS, 5 μl/100 mg), and were incubated at 37°C. Mortality rate was observed over 4 days post-infection.

In order to assess the bacterial load in the hemolymph, in a separate set of experiments, the larvae were infected with *S. aureus* as described above and then received either cinnamaldehyde (5.0 μg/100 g of larvae; *n* = 5/day) or vehicle (PBS; *n* = 5/day). Larvae were incubated at 37°C for up to 4 days. Five larvae of each group were culled per day and analyzed for bacterial load. Briefly, at each time point, the larvae were cut through in a cephalocaudal direction with a scalpel blade and squeezed to remove the hemolymph. Serial dilutions (10x) of the hemolymph of each larvae were made in PBS and 4 μl of each dilution were incubated in MHA and cultured for 24 h at 37°C. After this period, the plates were analyzed for CFU counting. The results are expressed as CFU/ml.

### Statistical Analysis

Statistical analyses were performed using the software GraphPad Prism version 5.0^1^. Data from were analyzed by two-way analysis of variance (ANOVA) and Tukey test. A *p*-value of <0.05 was considered as statistically significant. Differences in *G. mellonella* larvae survival were determined using the Kaplan–Meier method to calculate survival fractions and log-rank test was used to compare survival curves.

## Results

### Cinnamaldehyde Inhibits the Growth of *S. aureus* and *E. faecalis* without Inducing an Adaptive Phenotype

We initially analyzed the antimicrobial effects of cinnamaldehyde against clinical isolates and ATCC standard strains of *S. aureus* and *E. faecalis*. The strains showed different susceptibility profiles to clinically available antibiotics (**Table 1**). Amongst the three tested *E. faecalis* clinical isolates, two were resistant to at least one antibiotic: *E. faecalis* strain 2 (EF02) was resistant to penicillin (MAR index: 0.25) and *E. faecalis* strain 3 (EF03) was resistant to penicillin, gentamicin and ciprofloxacin (MAR index: 0.75). Of the four tested *S. aureus* strains, three were resistant to different antibiotics: *S. aureus* strains 2 (SA02) and 3 (SA03) were resistant to penicillin-oxacillin-clindamycin-sulfamethoxazole/trimethoprim (MAR index: 0.57) and *S. aureus* strain 4 (SA04) was resistant to penicillin-oxacillin-clindamycin (MAR index: 0.43).

Cinnamaldehyde was active against all strains of *E. faecalis* and *S. aureus*, including those with a multidrug resistance phenotype (**Table 2**). MIC values were of 0.25 mg/ml for all tested strains, except for the *S. aureus* standard strain ATCC 25923 (MIC value of 0.5 mg/ml). MBC values were of 1.0 mg/ml to all strains, 2–4-fold higher than each respective MIC, indicating a bactericidal action for cinnamaldehyde (**Table 2**). It is important to highlight that at the used concentration, the vehicle (2% DMSO in PBS) did not affect bacterial growth.

**Table 2:** Antimicrobial activity of cinnamaldehyde against *Staphylococcus aureus* and *Enterococcus faecalis.*

Strain	MIC^1^	MBC^2^
*E. faecalis* ATCC 19433	0.25	1
*E. faecalis* 1	0.25	1
*E. faecalis* 2	0.25	1
*E. faecalis* 3	0.25	1
*S. aureus* ATCC 25923	0.5	1
*S. aureus* ATCC 6538	0.25	1
*S. aureus* 1	0.25	1
*S. aureus* 2	0.25	1
*S. aureus* 3	0.25	1
*S. aureus* 4	0.25	1

*Experiments were performed three times in duplicate.*

*^1^Minimum Inhibitory Concentration (MIC) and ^2^Minimum Bactericidal Concentration (MBC) are expressed in mg/ml.*

Additionally, when incubated *in vitro* with cinnamaldehyde, neither *S. aureus* (ATCC 25923) nor *E. faecalis* (ATCC 19433) developed adaptive phenotypes even after 10 sequential passages. In contrast, both strains became tolerant to the clinically used antibiotic ciprofloxacin as MIC values increased from 0.0625 to 0.5 μg/ml for *S. aureus*, and from 0.125 to 0.5 μg/ml for *E. faecalis*.

### Cinnamaldehyde Sub-inhibitory Concentrations Do Not Affect Biofilm Formation by *E. faecalis*

**FIGURE 1:**
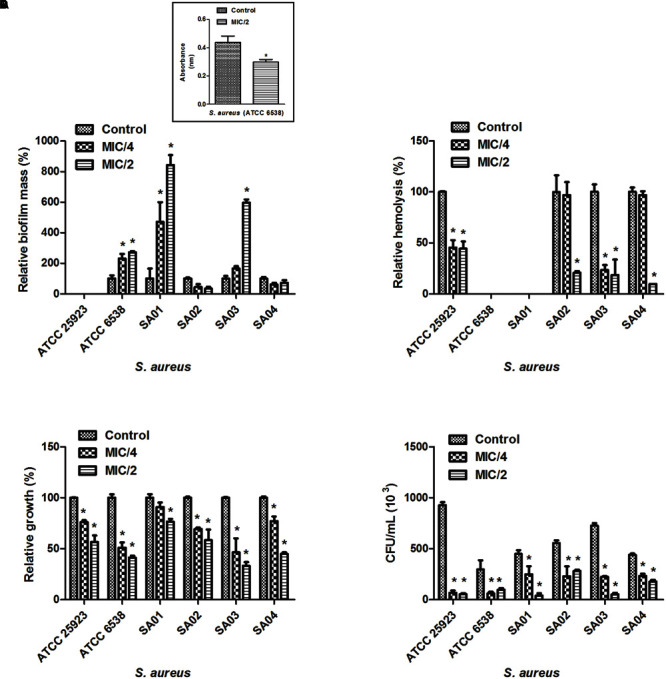
**Effect of cinnamaldehyde (MIC/2 and MIC/4) on virulence factors of *Staphylococcus aureus* strains. (A)** Biofilm mass production; **(B)** Hemolytic activity; **(C)** Serum resistance; **(D)** Adhesion to latex (catheter). Inset box indicates bacterial viability. ^∗^*p* < 0.05, compared with vehicle-treated controls. Experiments were performed three times in duplicate. Each bar represents mean + SD.

**FIGURE 2:**
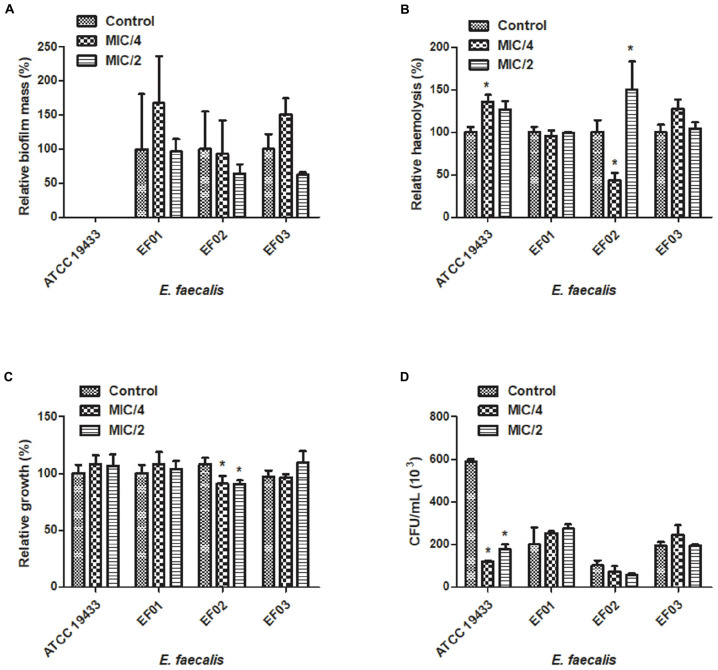
**Effect of cinnamaldehyde (MIC/2 and MIC/4) on virulence factors of *Enterococcus faecalis* strains. (A)** Biofilm mass production; **(B)** Hemolytic activity; **(C)** Serum resistance; **(D)** Adhesion to latex (catheter). ^∗^*p* < 0.05, compared with vehicle-treated controls. Experiments were performed three times in duplicate. Each bar represents mean + SD.

We attempted to analyze the effects of sub-inhibitory concentrations of cinnamaldehyde (MIC/4 or MIC/2) on the ability of *E. faecalis* and *S. aureus* to form biofilm. As depicted on **Figures 1A** and **2A**, cinnamaldehyde did not diminish biofilm formation by these bacteria at any of the tested concentrations. However, cinnamaldehyde treatment increased biofilm mass for some strains of *S. aureus* (*S. aureus* ATCC 6538, SA01 and SA03). In order to assess whether cinnamaldehyde-induced increase in biofilm formation is due to accumulation of dead cells, we evaluated the viability of the *S. aureus* (ATCC 6538) cells composing the biofilm. We found that cinnamaldehyde (MIC/2) decreases *S. aureus* viability (32.1 ± 3.9%; **Figure 1A**, inset box), indicating reduction in the number of viable cells.

### Cinnamaldehyde Sub-inhibitory Concentrations Inhibit the Hemolytic Activity of *S. aureus* but not *E. faecalis*

Three clinical isolates of *S. aureus* (SA02, SA03, SA04) and the standard *S. aureus* strain ATCC25923 were hemolytic. Hemolysis was reduced by cinnamaldehyde when tested at MIC/2 (*p* < 0.05) (**Figure 1B**). Percentage of inhibitions were of 99.9, 81.4, 90.3, and 55.7%, for SA02, SA03, SA04, and ATCC25923; respectively. The strains ATCC25923 and SA03 were also significantly inhibited (*p* < 0.05) by cinnamaldehyde at MIC/4 (54.4 and 76.7%, respectively). On the other hand, although all strains of *E. faecalis* caused hemolysis, only the strain EF02 was inhibited by cinnamaldehyde at the MIC/4 (56.5%; **Figure 2B**).

### Cinnamaldehyde Sub-inhibitory Concentrations Decrease *S. aureus* Survival in the Presence of Human Serum

We next determined whether cinnamaldehyde is able to enhance the lysis of *S. aureus* and *E. faecalis* in the presence of human serum. Our results show that when treated with cinnamaldehyde at MIC/2 and MIC/4, *S. aureus* strains were less able to survive when incubated with freshly isolated human serum (*p* < 0.05), except SA01 at MIC/4. Inhibitions ranged from 23.5% (SA01) to 66.9% (SA03) for cinnamaldehyde at MIC/2, and from 22.9% (SA01) to 53.5% (SA03) for cinnamaldehyde at MIC/4 (**Figure 1C**). Amongst the *E. faecalis* strains, only EF02 showed a slight reduction on its serum resistance when incubated with cinnamaldehyde (9.1 and 9.4% at MIC/4 and MIC/2, respectively) (**Figure 2C**).

### Cinnamaldehyde Sub-inhibitory Concentrations Decrease the Ability of *S. aureus* to Adhere to Latex

We also evaluated whether the sub-inhibitory concentrations of cinnamaldehyde were able to affect bacterial adhesion to latex, using a catheter model. As expected, all tested *S. aureus* and *E. faecalis* strains were able to adhere to latex. The sub-inhibitory concentrations of cinnamaldehyde were able to reduce the adherence to latex by all tested *S. aureus* strains (**Figure 1D**). When tested at MIC/2, cinnamaldehyde maximum inhibitory effects were observed for *S. aureus* ATCC 25923 (94.2%), and the clinical isolates SA03 (93.1%) and SA01 (91.3%). Also importantly, the same concentration of cinnamaldehyde diminished latex adhesion by *S. aureus* ATCC 6538 (67.4%), SA04 (59.6%), and SA02 (48.7%). The adhesion of *S. aureus* ATCC 25923 was also the most reduced by cinnamaldehyde at MIC/4 (93.0%), followed by SA01 (79.6%), SA03 (69.0%), SA02 (58.6%), SA04 (46.7%), and SA01 (44.7%). On the other hand, this compound only affected the adherence to latex of *E. faecalis* ATCC 19433 with reductions of 79.7 and 69.8% by cinnamaldehyde at MIC/2 and MIC/4, respectively (**Figure 2D**).

### Cinnamaldehyde Increases *G. mellonella* Larvae Survival and Reduces Bacterial Load in the Hemolymph

As cinnamaldehyde exhibited stronger actions on *S. aureus*, we performed an *in vivo* infection assay using *G. mellonella* larvae. Cinnamaldehyde or PBS (vehicle) did not induce any toxicity on larvae. Cinnamaldehyde (2.5–5.0 μg/100 mg of larvae) effects were compared to those of vehicle-treated larvae infected with *S. aureus* ATCC 25923. At 2 days post-infection, 80% of the vehicle-treated larvae had died, with no survivals on the third day.

**FIGURE 3:**
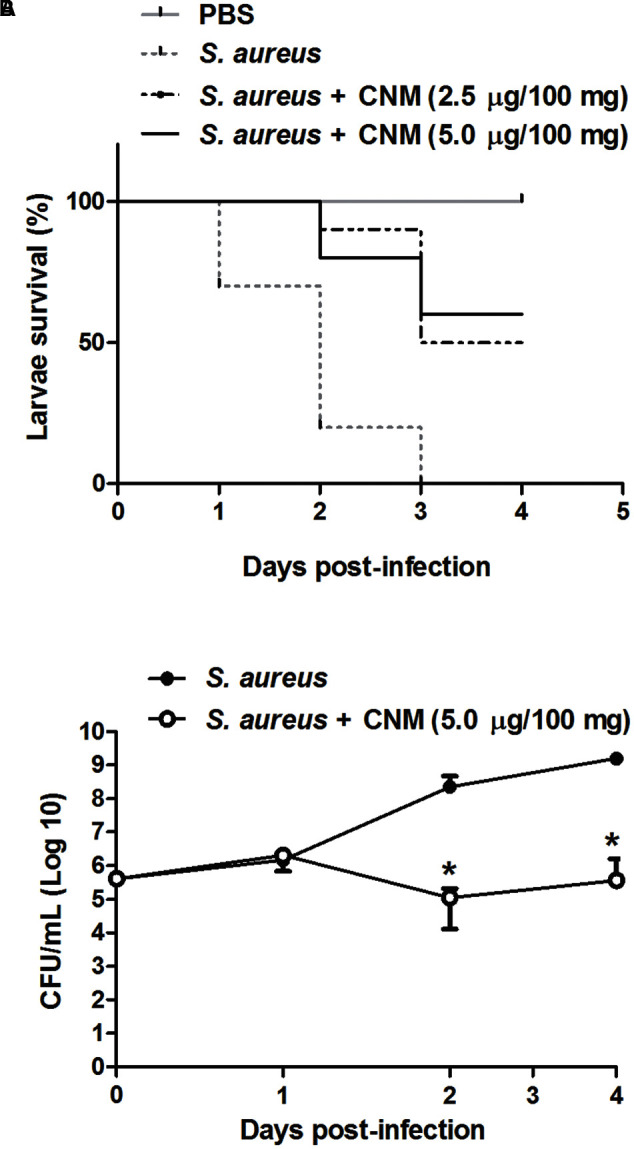
**Effect of cinnamaldehyde on survival (A)** and hemolymph bacterial load **(B)** of *Galleria mellonella* larvae infected with *S. aureus*. *G. mellonella* received either cinnamaldehyde (CNM; 2.5–5.0 μg/100 mg of larvae) or vehicle (PBS, 5 μl/100 mg) and were evaluated for 4-days post-infection. *n* = 10/group for survival experiments; *n* = 5/group/day for bacterial load quantification. Data for bacterial load is represented as mean + SD. ^∗^*p* < 0.05, compared with vehicle-treated larvae. Experiments were performed twice in duplicate.

In contrast, cinnamaldehyde enhanced *G. mellonella* larvae survival, as >50% remained alive on day 4 (*p* < 0.05; **Figure 3A**). This effect was more pronounced for the highest dose of the compound (5.0 μg/100 mg of larvae). By analyzing the bacterial load, it was observed that cinnamaldehyde significantly reduces the number of *S. aureus* in *G. mellonella* hemolymph samples in comparison with vehicle-treated larvae, as indicated by CFU counting (4-log reduction in bacterial survival). This effect was noted from 48 h post-infection and remained significant throughout the rest of the assay (**Figure 3B**).

## Discussion

### Cinnamaldehyde Inhibits the Growth of *S. aureus* and *E. faecalis* without Inducing an Adaptive Phenotype

Cinnamaldehyde presented with antimicrobial actions on clinical isolates of *S. aureus* and *E. faecalis*, in addition to ATCC standard strains. This compound was effective on all strains of *E. faecalis* and *S. aureus*, including those with a multidrug resistance phenotype. The antimicrobial properties of cinnamaldehyde have been demonstrated against a range of Gram-positive and Gram-negative pathogens including *S. aureus* and *E. faecalis* (Cox and Markham, 2007; Shen et al., 2015; Upadhyay and Venkitanarayanan, 2016). Cinnamaldehyde actions against these pathogens are related to changes in their cell membrane polarity and permeability (Hammer and Heel, 2012). Importantly, we show for the first time that although becoming tolerant to ciprofloxacin, neither *S. aureus* (ATCC 25923) or *E. faecalis* (ATCC 19433) develop an adaptive phenotype when incubated with cinnamaldehyde *in vitro*.

### Cinnamaldehyde Sub-inhibitory Concentrations Do Not Affect Biofilm Formation by *E. faecalis*

Biofilm formation is an important virulence factor involved in staphylococcal and enterococcal infections (Claessen et al., 2014). In a recent report, cinnamaldehyde was shown to inhibit the expression of *sarA* (a positive regulator of biofilm formation) in *S. aureus* at sub-inhibitory concentrations (Jia et al., 2011). Surprisingly, cinnamaldehyde not only had no effects on the ability of *E. faecalis* to form biofilm, but enhanced biofilm formation by some strains of *S. aureus*. These results oppose to those of previously published reports in that this compound was suggested to reduce biofilm formation by these bacteria. Recently, Budri et al. (2015) showed that cinnamaldehyde strongly diminishes biofilm formation by *S. aureus* ATCC 35983 on both polystyrene and stainless steel surfaces, when tested at 0.199 mg/ml. This effect was also observed when cinnamaldehyde was associated with either biodegradable polymers or nanoparticles (Zodrow et al., 2012; Duncan et al., 2015). Similarly, a *Cinnamomum zeylanicum* essential oil, rich in cinnamaldehyde, was shown to reduce biofilm formation by *E. faecalis* (Abbaszadegan et al., 2016). It is possible that the discrepancies found between our results and the above discussed are due to differences on the strains tested which may present different virulence patterns.

Additionally, different antimicrobial agents may be able to induce biofilm formation at sub-inhibitory concentrations (Kaplan et al., 2012; Schilcher et al., 2016). This effect is suggested to be strain-specific and related to the induction of stress pathways in *S. aureus* that in turn, lead to the expression of biofilm-associated genes (Schilcher et al., 2016). Herein, as in other reports (Kaplan et al., 2012; Schilcher et al., 2016), biofilm formation was evaluated by the crystal violet assay. This method is widely used for this purpose, however, crystal violet stains both viable and dead cells, in addition to the extracellular matrix (Xu et al., 2016). In order to determine whether biofilm formation by *S. aureus* was associated with cell survival, we evaluated the viability of biofilm-forming *S. aureus* (ATCC 6538) cells incubated with cinnamaldehyde at MIC/2. We found that at this concentration, cinnamaldehyde reduces *S. aureus* metabolic activity, indicating loss of viable cells. These results allow us to suggest that the increased biofilm mass observed in cinnamaldehyde-treated *S. aureus* is due to the accumulation of dead cells rather than increase in *S. aureus* virulence. They also support the applicability of cinnamaldehyde in the treatment of *S. aureus*-induced infections.

### Cinnamaldehyde Sub-inhibitory Concentrations Inhibit the Hemolytic Activity of *S. aureus* but not *E. faecalis*

Hemolytic toxins are secreted virulence factors expressed by some strains of *S. aureus* and *E. faecalis* which increase pathogenicity (Van Tyne et al., 2013; Tabor et al., 2016). Our data show that cinnamaldehyde diminishes *S. aureus*-induced hemolysis, but is only able to inhibit this parameter in one of the tested *E. faecalis* strains. The effects of cinnamaldehyde on cell survival have been widely studied in different cells lines, including immune cells (Roth-Walter et al., 2014), neurones (Pyo et al., 2013), erythrocytes (Theurer et al., 2013), amongst others. Of importance, this compound was shown to cause hemolysis *per se* when incubated for 48 h with human erythrocytes (Theurer et al., 2013). On the other hand, we show that the hemolysis caused by *S. aureus* is markedly inhibited by sub-inhibitory concentrations of cinnamaldehyde. It is possible that in this experimental setting, cinnamaldehyde targets bacteria rather than erythrocytes. Similarly, Amalaradjou et al. (2014) reported a protective effect for cinnamaldehyde in *Cronobacter sakazakii*-induced intestinal epithelial cell death.

### Cinnamaldehyde Sub-inhibitory Concentrations Decrease *S. aureus* Survival in the Presence of Human Serum

*Staphylococcus aureus* and *E. faecalis* are common etiological agents of bacteraemia which often lead to septic shock and endocarditis (Dahl et al., 2016; Yahav et al., 2016). The survival of these pathogens in the bloodstream is due to their ability to express different virulence factors that target components of the host’s immune system (Foster et al., 2014; Hall et al., 2015; Richards et al., 2015). We show that cinnamaldehyde enhances *S. aureus* but not *E. faecalis* killing when incubated with freshly isolated human serum. To the best of our knowledge, this study presents the first evidence on that cinnamaldehyde impairs *S. aureus* resistance to human serum. This may represent an additional mechanism by which cinnamaldehyde confers protection to infection *in vivo*.

### Cinnamaldehyde Sub-inhibitory Concentrations Decrease the Ability of *S. aureus* to Adhere to Latex

Catheterization is a potential risk factor for bacterial colonization and infection (Padmavathy et al., 2015; Tong et al., 2015). *S. aureus* and *E. faecalis* are both capable of adhering to abiotic surfaces (such as catheter) due to the expression of surface proteins (Foster et al., 2014), such as the *S. aureus* protein A (SpA) and the enterococcal surface protein (Esp) (Elhadidy and Elsayyad, 2013; Zapotoczna et al., 2016). Cinnamaldehyde strongly inhibited *S. aureus* adherence to latex, an effect that was observed when this compound was tested at MIC/2 and MIC/4 on all strains. When tested on *E. faecalis* strains, cinnamaldehyde only diminished latex adherence by the standard strain ATCC 19433.

The use of essential oils or other plant-derived material to prevent bacterial adhesion to catheters and other medical devices has been pointed as an interesting approach for the medical field (Silva et al., 2016). These strategies involve the modification of the surface by the incorporation of the anti-adhesive compounds, resulting in functionalized surfaces with improved resistance to microbial colonization (Grumezescu, 2013; Trentin et al., 2015). Our results show that cinnamaldehyde may be useful for the development of surface-modified materials in order to prevent *S. aureus* adhesion.

### Cinnamaldehyde Increases the *G. mellonella* Larvae Survival and Reduce Bacterial Load in the Hemolymph

Overall, cinnamaldehyde *in vitro* antimicrobial actions were more pronounced on *S. aureus*. *In vivo*-testing of cinnamaldehyde in *G. mellonella* larvae infected with *S. aureus* showed this compound augments larvae survival whilst reducing bacterial load in their hemolymph. *In vivo* protection of infection by cinnamaldehyde has been previously reported. Recently, cinnamaldehyde was shown to protect *G. mellonella* larvae against *L. monocytogenes*-induced infection (Upadhyay and Venkitanarayanan, 2016). This action was attributed to its ability to up-regulate the expression of antimicrobial peptide genes in *G. mellonella* (Upadhyay and Venkitanarayanan, 2016). The immunomodulatory properties of cinnamaldehyde were also evaluated in a mouse model of LPS-induced SIRS. The authors showed that cinnamaldehyde protection is related to the ability of this compound in modulating the immune response through transient receptor potential ankyrin 1 (TRPA1)-dependent and independent mechanisms (Mendes et al., 2016).

These results are rather promising as cinnamaldehyde is effective against bacteria and also improves the immune response to infection by these pathogens. Here, cinnamaldehyde protected against *S. aureus* infection in *G. mellonella*, in doses equivalent to 25–50 mg/kg. On the other hand, further studies are necessary in order to establish cinnamaldehyde safety and effectiveness in humans when given by oral route. Indeed, reports suggest that cinnamaldehyde presents both genotoxic and irritative effects, although these are noted when this compound is administered at much higher concentrations/doses than the ones investigated in our study, such as >500 mg/kg (systemically) or >3% (topically applied to the skin) (for review see, Bickers et al., 2005). Data obtained from animal studies suggest cinnamaldehyde is safe by oral route when administered as either a single dose (2,220 mg/kg) or repeatedly for even over 2 years (up to 550 mg/kg/day). Importantly, cinnamaldehyde excretion rate at 24 h after administration varies between 70 and 98% in rodents, depending on the route of administration; and reaches 100% within 8 h when given orally to healthy human volunteers (for review see, Bickers et al., 2005; Cocchiara et al., 2005). Thus, implementation of dose schemes may also consider the excretion rate of cinnamaldehyde.

Overall, we suggest that cinnamaldehyde may represent an alternative therapy to control *S. aureus*-induced bacterial infections as it presents the ability to diminish bacterial virulence/survival in addition to improve the host’s immune response to infection.

## Author Contributions

TF, JA, BdSP, JdS, ES, BdS, VC, TN, CF, CS, JC, VM-N, LdS, and EF contributed to conception, design, data acquisition, analysis, and interpretation, drafted and critically revised the manuscript. All authors gave final approval and agree to be accountable for all aspects of the work.

## Conflict of Interest Statement

The authors declare that the research was conducted in the absence of any commercial or financial relationships that could be construed as a potential conflict of interest.

## Footnotes

1 www.graphpad.com
