# Corrigendum: Cinnamaldehyde Inhibits *Staphylococcus aureus* Virulence Factors and Protects against Infection in a *Galleria mellonella* Model

**DOI:** 10.3389/fmicb.2020.628074

**Published:** 2021-01-15

**Authors:** Thiago A. F. Ferro, Jéssica M. M. Araújo, Bruna L. dos Santos Pinto, Jéssica S. dos Santos, Eliene B. Souza, Bruna L. R. da Silva, Valderlane L. P. Colares, Tânia M. G. Novais, Clovis M. B. Filho, Carsten Struve, João B. Calixto, Valério Monteiro-Neto, Luís C. N. da Silva, Elizabeth S. Fernandes

**Affiliations:** ^1^Programa de Pós-graduação, Universidade CEUMA, São Luís, Brazil; ^2^Universidade Federal de Pernambuco, Pernambuco, Brazil; ^3^Statens Serum Institut, Copenhagen, Denmark; ^4^Centro de Inovação e Estudos Pré-clínicos, Florianópolis, Brazil; ^5^Universidade Federal Do Maranhão, São Luís, Brazil

**Keywords:** essential oil, cinnamaldehyde, infection, bacterial virulence, *S. aureus*

In the original article, there were mistakes in Tables 1 and 2, and Figure 2 as published. Instead of the accession ATCC 19443 for the type strain of *E. faecalis*, it should read ATCC 19433 in Tables 1 and 2, and Figure 2, **as well as throughout the text (in** ***MATERIALS*** ***AND METHODS*, section** ***Bacterial Strains***, ***1st paragraph and section Analysis of*** ***Bacterial Tolerance to Drug***, ***1st paragraph; in RESULTS***, section ***Cinnamaldehyde*** ***Inhibits the Growth of S. aureus and E. faecalis without Inducing an Adaptive*** ***Phenotype***, ***3rd paragraph, section Cinnamaldehyde Sub-inhibitory Concentrations*** ***Decrease the Ability of S. aureus to Adhere to Latex***, ***1st paragraph; and in the*** ***DISCUSSION***, ***section Cinnamaldehyde Inhibits the Growth of S. aureus and E. faecalis*** ***without Inducing an Adaptive Phenotype***, ***1st paragraph, section Cinnamaldehyde*** ***Sub-inhibitory Concentrations Decrease the Ability of S. aureus to Adhere to Latex***, ***1st*** ***paragraph)*.**

**Table 1:** Antibiotic susceptibility profiles of *Enterococcus faecalis* and *Staphylococcus aureus* strains.

**Strain**	**Antibiotic**							**MAR**
	**PEN**	**VAN**	**OXA**	**GEN**	**CLI**	**CIP**	**SUT**	
*E. faecalis* ATCC 19433	S	S	–	S	–	S	–	0
*E. faecalis* 1	S	S	–	S	–	S	–	0
*E. faecalis* 2	R	S	–	S	–	S	–	0.25
*E. faecalis* 3	R	S	–	R	–	R	–	0.75
*S. aureus* ATCC 25923	S	S	S	S	S	S	S	0
*S. aureus* ATCC 6538	S	S	S	S	S	S	S	0
*S. aureus* 1	S	S	S	S	S	S	S	0
*S. aureus* 2	R	S	R	S	R	S	R	0.50
*S. aureus* 3	R	S	R	S	R	S	R	0.50
*S. aureus* 4	R	S	R	S	R	S	S	0.43

*Experiments were performed three times in duplicate*.

*PEN, Penicillin; VAN, vancomycin; OXA, oxacillin; GEN, gentamicin; CLI, clindamycin, CIP, ciprofloxacin; SUT, sulfamethoxazole/trimethoprim. Multiple antibiotic resistance (MAR) index*.

**Table 2:** Antimicrobial activity of cinnamaldehyde against *Staphylococcus aureus* and *Enterococcus faecalis*.

**Strain**	**MIC^1^**	**MBC^2^**
*E. faecalis* ATCC 19433	0.25	1
*E. faecalis* 1	0.25	1
*E. faecalis* 2	0.25	1
*E. faecalis* 3	0.25	1
*S. aureus* ATCC 25923	0.5	1
*S. aureus* ATCC 6538	0.25	1
*S. aureus* 1	0.25	1
*S. aureus* 2	0.25	1
*S. aureus* 3	0.25	1
*S. aureus* 4	0.25	1

*Experiments were performed three times in duplicate*.

1 Minimum Inhibitory Concentration (MIC) and

2 *Minimum Bactericidal Concentration (MBC) are expressed in mg/ml*.

**Figure 2:**
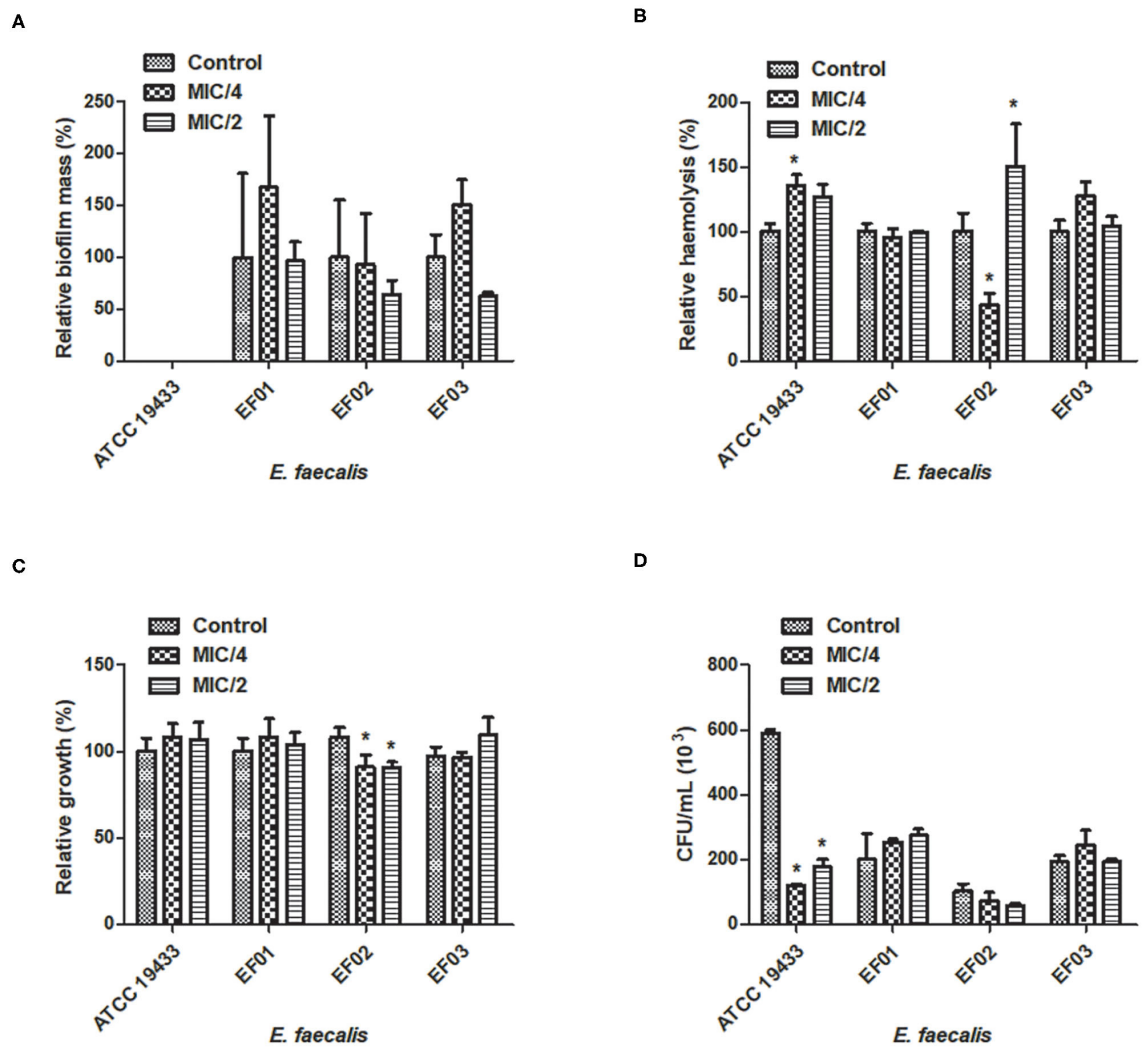
Effect of cinnamaldehyde (MIC/2 and MIC/4) on virulence factors of *Enterococcus faecalis* strains. **(A)** Biofilm mass production; **(B)** Haemolytic activity; **(C)** Serum resistance; **(D)** Adhesion to latex (catheter). ^*^p < 0.05, compared with vehicle-treated controls. Experiments were performed three times in duplicate. Each bar represents mean ± SD.

In the original article, there was an error. All instances of *E. faecalis* ATCC 19443 should read ATCC 19433.

Corrections has been made to the following sections:

***MATERIALS AND METHODS***, ***Bacterial Strains***, ***1st paragraph***:

All tested bacteria were kindly provided by the bacterial collection sector of the Universidade CEUMA and included: six strains of *S. aureus* (standard strains ATCC 25923 and ATCC 6538; clinical isolates SA01, SA02, SA03, SA04); four strains of *E. faecalis* (standard strain ATCC 19433; clinical isolates EF01, EF02, EF03). Susceptibility to antimicrobials was determined in an automated VITEK® 2 system (BioMérieux Clinical Diagnostics, USA) and data interpretation was performed as recommended by the Clinical Laboratory Standards Institute (2015). The multiple antibiotic resistance (MAR) index was calculated using the formula MAR = x/y, where “x” was the number of antibiotics to which the isolate demonstrated resistance; and “y” was the total number of antibiotics tested. The antibiotic susceptibility profile of each strain is shown at Table 1.

***MATERIALS AND METHODS***, ***Analysis of Bacterial Tolerance to Drug***, ***1st*** ***paragraph***:

In order to investigate whether cinnamaldehyde is able to induce bacterial tolerance to drug, we performed serial passage experiments, using the standard strains of *S. aureus* (ATCC 25923) and *E. faecalis* (ATCC 19433). For this, bacterial suspensions (1 ml, ~1.5 × 10^8^ CFU/ml) were added to six-well tissue culture plates containing MH broth and sub-inhibitory concentrations (MIC/2) of cinnamaldehyde or ciprofloxacin (positive control). After 24 h at 37°C, the culture growing at one dilution below the MIC was used to inoculate the subsequent passage, and this process was repeated for a total of 10 passages. The compound concentration range of each new passage was based on the MIC calculated for the previous passage. Vehicle-treated bacteria (2% DMSO in PBS) were used as negative controls.

***RESULTS***, ***Cinnamaldehyde Inhibits the Growth of S. aureus and E. faecalis without*** ***Inducing an Adaptive Phenotype***, ***3rd paragraph***:

Additionally, when incubated *in vitro* with cinnamaldehyde, neither *S. aureus* (ATCC 25923) nor *E. faecalis* (ATCC 19433) developed adaptive phenotypes even after 10 sequential passages. In contrast, both strains became tolerant to the clinically used antibiotic ciprofloxacin as MIC values increased from 0.0625 μg/ml to 0.5 μg/ml for *S. aureus*, and from 0.125 μg/ml to 0.5 μg/ml for *E. faecalis*.

***RESULTS***, ***Cinnamaldehyde Sub-inhibitory Concentrations Decrease the Ability of S*.** ***aureus to Adhere to Latex***, ***1st paragraph***:

We also evaluated whether the sub-inhibitory concentrations of cinnamaldehyde were able to affect bacterial adhesion to latex, using a catheter model. As expected, all tested *S. aureus* and *E. faecalis* strains were able to adhere to latex. The sub-inhibitory concentrations of cinnamaldehyde were able to reduce the adherence to latex by all tested *S. aureus* strains (Figure 1D). When tested at MIC/2, cinnamaldehyde maximum inhibitory effects were observed for *S. aureus* ATCC 25923 (94.2%), and the clinical isolates SA03 (93.1%) and SA01 (91.3%). Also importantly, the same concentration of cinnamaldehyde diminished latex adhesion by *S. aureus* ATCC 6538 (67.4%), SA04 (59.6%), and SA02 (48.7%). The adhesion of *S. aureus* ATCC 25923 was also the most reduced by cinnamaldehyde at MIC/4 (93.0%), followed by SA01 (79.6%), SA03 (69.0%), SA02 (58.6%), SA04 (46.7%), and SA01 (44.7%). On the other hand, this compound only affected the adherence to latex of *E. faecalis* ATCC 19433 with reductions of 79.7 and 69.8% by cinnamaldehyde at MIC/2 and MIC/4, respectively (Figure 2).

***DISCUSSION***, ***Cinnamaldehyde Inhibits the Growth of S. aureus and E. faecalis*** ***without Inducing an Adaptive Phenotype***, ***1st paragraph***:

Cinnamaldehyde presented with antimicrobial actions on clinical isolates of *S. aureus* and *E. faecalis*, in addition to ATCC standard strains. This compound was effective on all strains of *E. faecalis* and *S. aureus*, including those with a multidrug resistance phenotype. The antimicrobial properties of cinnamaldehyde have been demonstrated against a range of Gram-positive and Gram-negative pathogens including *S. aureus* and *E. faecalis* (Cox and Markham, 2007; Shen et al., 2015; Upadhyay and Venkitanarayanan, 2016). Cinnamaldehyde actions against these pathogens are related to changes in their cell membrane polarity and permeability (Hammer and Heel, 2012). Importantly, we show for the first time that although becoming tolerant to ciprofloxacin, neither *S. aureus* (ATCC 25923) or *E. faecalis* (ATCC 19433) develop an adaptive phenotype when incubated with cinnamaldehyde *in vitro*.

***DISCUSSION***, ***Cinnamaldehyde Sub-inhibitory Concentrations Decrease the Ability of*** ***S. aureus to Adhere to Latex***, ***1st paragraph***:

Catheterization is a potential risk factor for bacterial colonization and infection (Padmavathy et al., 2015; Tong et al., 2015). *S. aureus* and *E. faecalis* are both capable of adhering to abiotic surfaces (such as catheter) due to the expression of surface proteins (Foster et al., 2014), such as the *S. aureus* protein A (SpA) and the enterococcal surface protein (Esp) (Elhadidy and Elsayyad, 2013; Zapotoczna et al., 2016). Cinnamaldehyde strongly inhibited *S. aureus* adherence to latex, an effect that was observed when this compound was tested at MIC/2 and MIC/4 on all strains. When tested on *E. faecalis* strains, cinnamaldehyde only diminished latex adherence by the standard strain ATCC 19433.

The authors apologize for these errors and state that this does not change the scientific conclusions of the article in any way. The original article has been updated.
